# Synthesis, Structural Characterization, and Infrared Analysis of Double Perovskites Pr_2_NiMnO_6_, Gd_2_NiMnO_6_, and Er_2_NiMnO_6_ Functional Nano-Ceramics

**DOI:** 10.3390/nano14110960

**Published:** 2024-05-30

**Authors:** Mebark Elhamel, Zoulikha Hebboul, Djamal Benbertal, Pablo Botella, Daniel Errandonea

**Affiliations:** 1 Laboratoire Physico-Chimie des Matériaux, Université Amar Telidji de Laghouat, BP 37G, Route de Ghardaia, Laghouat 03000, Algeria; m.elhamel@lagh-univ.dz (M.E.); d.benbertal1932@yahoo.com (D.B.); 2 Departamento de Física Aplicada—ICMUV, MALTA Consolider Team, Universidad de Valencia, Edificio de Investigación, Carrer del Doctor Moliner 50, 46100 Burjassot, Valencia, Spain; pablo.botella-vives@uv.es

**Keywords:** sol–gel method, Pr_2_NiMnO_6_ nano-ceramic, XRD, FTIR

## Abstract

We synthesized Pr_2_NiMnO_6_, Gd_2_NiMnO_6_, and Er_2_NiMnO_6_ double perovskites in a nano-ceramic form by a sol–gel method. By means of room-temperature X-ray powder diffraction measurements, we determined the crystal structure of the three compounds, which is monoclinic, corresponding to a double perovskite structure, described by space group *P*2_1_/*n* structure. From the determined structures, the bulk moduli were estimated to be 173–179 GPa. The average size particle of nanoparticles was determined from X-ray diffraction by the Langford method plot and by the Scherrer formula. The morphology and homogeneity of nanoparticles were analyzed by scanning electron microscopy. We found that they form compact agglomerations of approximately 200 nm in diameter. Fourier transform infrared spectroscopy measurements were performed, determining the absorption spectrum. The assignment of the measured infrared absorption bands is discussed.

## 1. Introduction

Double perovskites expand the chemical and structural space of the perovskite family and have emerged as promising alternatives with improved catalytic performance and many other interesting properties for technological applications [1,2]. Double perovskite R_2_NiMnO_6_ compounds, where R is a lanthanide element, are materials that stand out for electronic interaction which give rise to a ferromagnetic alignment of *d* electron spins, due to the super-exchange interaction between Mn^4+^ and Ni^2+^ ions according to the Goodenough–Kanamori rules [3]. These materials have recently received increased interest because of their unique optical, electronic, and magnetic properties [4,5]. They have also attracted much attention due to their potential use in magnetic cooling, environmentally friendly technologies based on the magnetocaloric effect [6,7,8,9]. It has been also proposed that, thanks to their complex physical properties, R_2_NiMnO_6_ compounds may be used to design a variety of innovative devices, including multiple-state memory elements [10]. The discovery of the existence of a magnetodielectric effect around the ferromagnetic Curie temperature of compounds like La_2_NiMnO_6_ represented a step forward for these developments [11]. R_2_NiMnO_6_ compounds are also ferromagnetic semiconductors, being therefore promising materials for spintronic devices for energy-efficient data storage and energy harvesting [12]. These materials also exhibit magnetocapacitance and magnetoresistance near room temperature [13] and have been used for improving the performance of solid oxide fuel cells [14], which adds more value to R_2_NiMnO_6_ compounds as multifunctional materials. Another interesting fact in lanthanide double perovskites is related to the lanthanide contraction in ionic radius and gadolinium break in ionic electronegativity. These facts have been found to regulate the properties in the perovskite structure, including the catalytic performance [15]. Thus, R_2_NiMnO_6_ compounds have direct interest from both fundamental and technological points of view. More information on the multiple recent developments in the study of R_2_NiMnO_6_ double perovskite can be found in a recently published review [16].

The prospective of technological utilization of the multifunctional properties of R_2_NiMnO_6_ compounds could be boosted by the synthesis of these materials as nanoparticles [17]. Nanomaterials have currently a considerable impact in practically all domains of science and technology because, thanks to size effects, the properties of materials, such as reactivity, strength, and electrical and optical characteristics, can be tuned for different applications [18]. Polycrystalline R_2_NiMnO_6_ double perovskite compounds are usually synthesized by conventional solid-state methods [6], by low-temperature auto-ignition methods [19], or by annealing a compact mixture of R_2_O_3_, NiO, and MnO_2_ under high pressure using a belt-type apparatus [20]. They have been also prepared from citrate precursors obtained by a soft-chemistry procedure [17]. 

In this work, we present a simple, cheap, and environmentally friendly soft-chemistry method to prepare nanoparticles of Pr_2_NiMnO_6_, Gd_2_NiMnO_6_, and Er_2_NiMnO_6_. This is the first time nanoparticles of the studied compounds are reported. To the best of the authors knowledge, the used method has never been used before to prepare R_2_NiMnO_6_ compounds. The morphology, crystal structure, and infrared transmittance of the samples were characterized via scanning electron microscopy (SEM), powder X-ray diffraction (XRD), and Fourier transform infrared (FTIR) spectroscopy. The bulk modulus and important elastic modulus have been also estimated. The size of the obtained particles ranges from 29–36 nm to 40–49 nm in the different compounds according to the two methods used for determining the particle size.

## 2. Materials and Methods

All the reagents used in the synthesis were purchased from Sigma–Aldrich (St. Louis, MO, USA) with 99.99% of purity. They include nickel nitrate hexahydrate (Ni(NO_3_)_2_·6H_2_O), praseodymium(III) nitrate hexahydrate (Pr(NO_3_)_3_·6H_2_O), erbium(III) oxide (Er_2_O_3_), gadolinium nitrate hexahydrate (Gd(NO_3_)_3_·6H_2_O), and manganese chloride tetrahydrate (MnCl_2_·4H_2_O). The samples R_2_NiMnO_6_, R = Pr, Gd, and Er, as nanoparticles, were prepared by sol–gel synthesis. The weights of all reagents and reaction conditions are summarized in Table 1. For each sol preparation, MnCl_2_·4H_2_O and Ni(NO_3_)·6H_2_O were mixed in methanol for 10 min using a magnetic mixer; then, Pr(NO_3_)_3_·6H_2_O, Er_2_O_3_, and Gd(NO_3_)_3_·6H_2_O were added separately to the independent solutions and mixed for another 20, 10, and 10 min, respectively. After that, a solution of citric acid (C_6_H_8_O_7_), adjusting the solution pH to 6.5, was added to the mixture and heating was kept at 70 °C until gel formation at 22, 48, and 32 min, respectively. The three gels were dried at 80 °C in a cabinet drier for 24 h to obtain precursor powders. Then, an agate mortar and pestle were used to grind the mixture prepared for each sample. Subsequently pellets were prepared under a pressure of ~45 bars and were subsequently annealed at 1100 °C for 3 h under air atmosphere. 

Sample morphology, homogeneity, and chemical composition of the obtained products were analyzed by scanning SEM and energy-dispersive X-ray analysis (EDX) using a Tescan Vega (Brno, Czech Republic) scanning electron microscope connected to a Peltier cooled XFlashTM Bruker (Billerica, MA, USA) silicon drift detector and using an accelerating voltage of 30 kV. Crystal structures were characterized by powder XRD using a Panalytical EMPYREAN diffractometer (Malvern, UK) operating in a Bragg–Brentano geometry (Cu K_α1_, 40 mA, 30 kV) with a 0.01° step size and an acquisition time of 6 s/step in the 10−90° 2θ range. FTIR spectra were recorded in transmission mode using a Jasco FT/IR-4200 (Tokyo Japan) set up using ultrapure KBr pellets. 

## 3. Results and Discussion

### 3.1. Morphology

**Figure 1:**
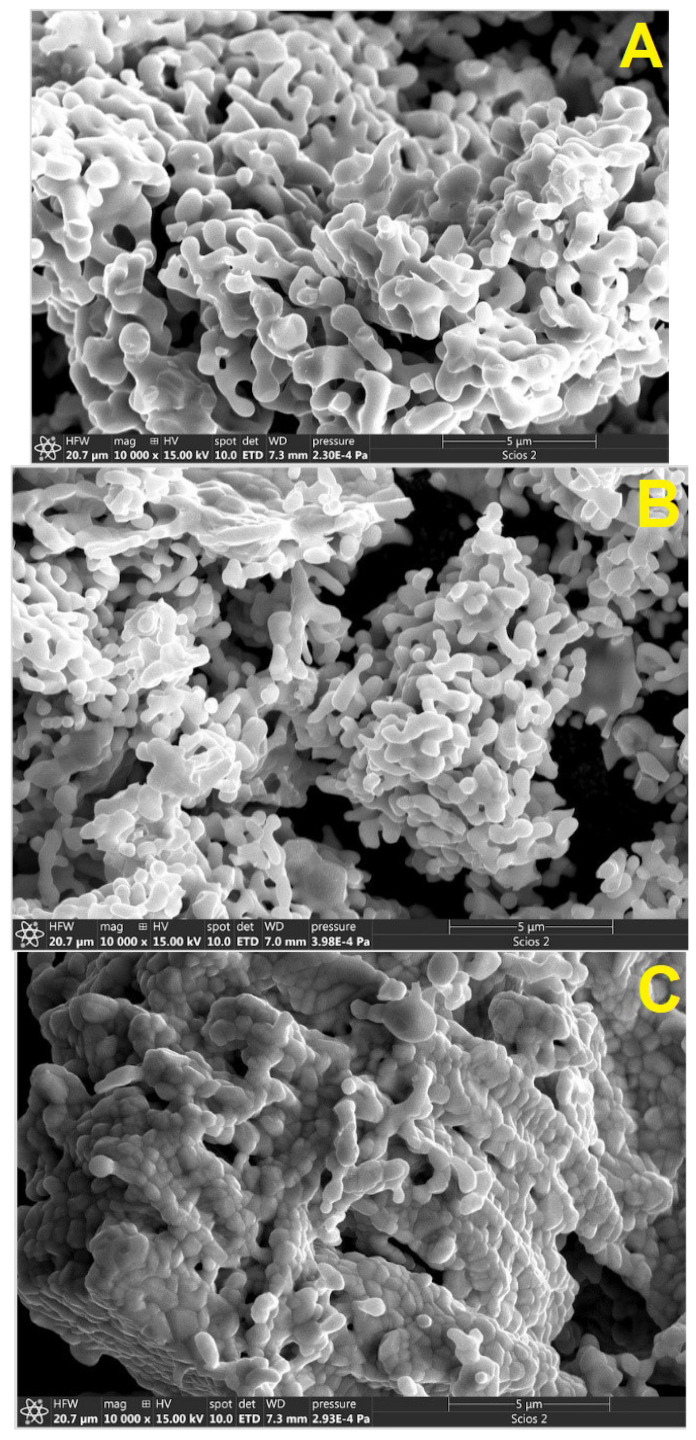
SEM images of agglomerations of synthesized nanoparticles. (**A**) Pr_2_NiMnO_6_, (**B**) Gd_2_NiMnO_6_, and (**C**) Er_2_NiMnO_6_.

SEM images of the synthesized samples illustrating their microstructure are shown in Figure 1. The micrographs notably show that the three samples have a similar homogeneous morphology, which consisted of quasi-spherical nanoparticles having uniform grain sizes and distributions throughout the three samples. Figure 1 also shows that R_2_NiMnO_6_ nanoparticles are arranged sequentially as chains, forming strongly bound agglomerations and leading to submicron-sized entities. The average diameter of agglomerations is between 100 and 200 nm in diameter. In the case of Pr_2_NiMnO_6_ and Gd_2_NiMnO_6,_ the agglomerations bear a resemblance to zeolite-type structures. In the case of Er_2_NiMnO_6_, the agglomerations look like stacked coral reefs. The differences on the shape of agglomerations might be related to the different concentration of impurities of the samples (see discussion of XRD). As a consequence of the strong agglomeration between nanoparticles, we could not determine the particle of nanoparticles from SEM measurements. The particle size was determined from XRD. The mean chemical compositions of the synthesized samples were analyzed by EDX analyses. We found within experimental uncertainties that the composition of the samples, by both weight percent and atomic percent of Pr/Gd/Er, Ni, and Mn, were consistent with their corresponding nominal compositions of 2:1:1. The readers should note that, in this manuscript, we do not characterize and analyze the materials’ lattice oxygen and oxygen vacancies, which might be important for oxides [21]. Future studies using O 1s XPS or chemical titration are needed to obtain such information, but they are beyond the scope of the present study.

### 3.2. XRD Analysis

**Figure 2:**
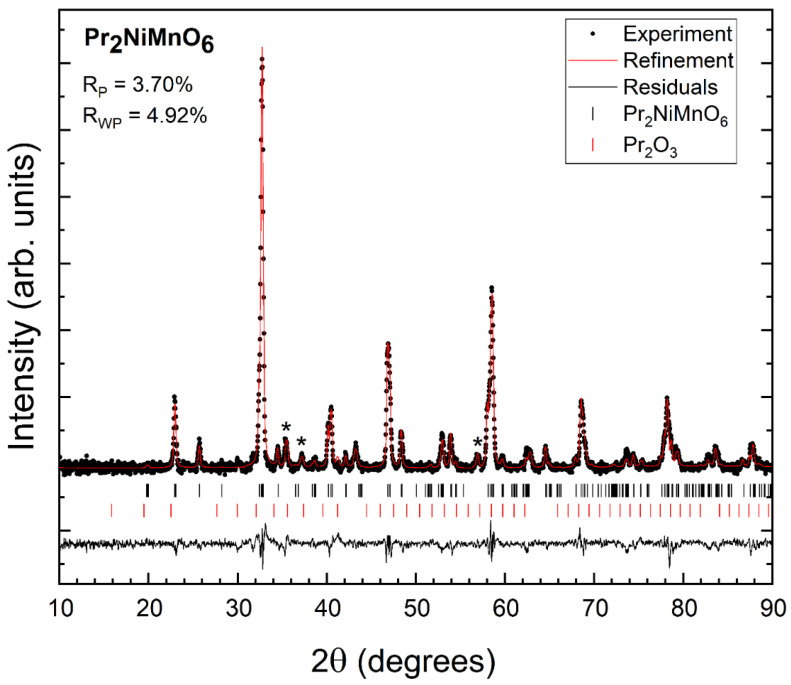
XRD pattern measured in Pr_2_NiMnO_6_ (black symbols), Rietveld refinement (red line), and residuals (black line). The ticks show the position of the calculated peaks. R-values are provided in the figure. Asterisks are the most intense peaks of the minority phase.

**Figure 3:**
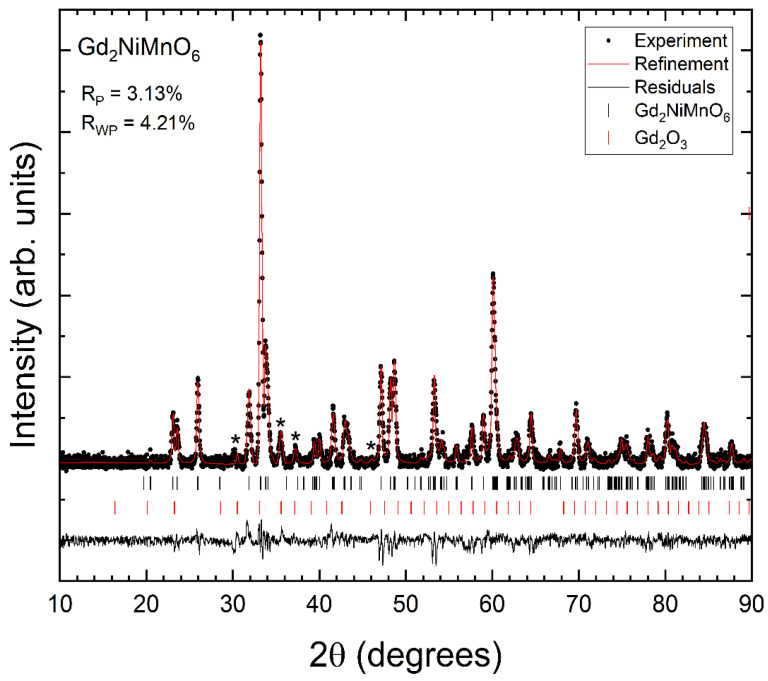
XRD pattern measured in Gd_2_NiMnO_6_ (black symbols), Rietveld refinement (red line), and residuals (black line). The ticks show the position of the calculated peaks. R-values are provided in the figure. Asterisks are the most intense peaks of the minority phase.

**Figure 4:**
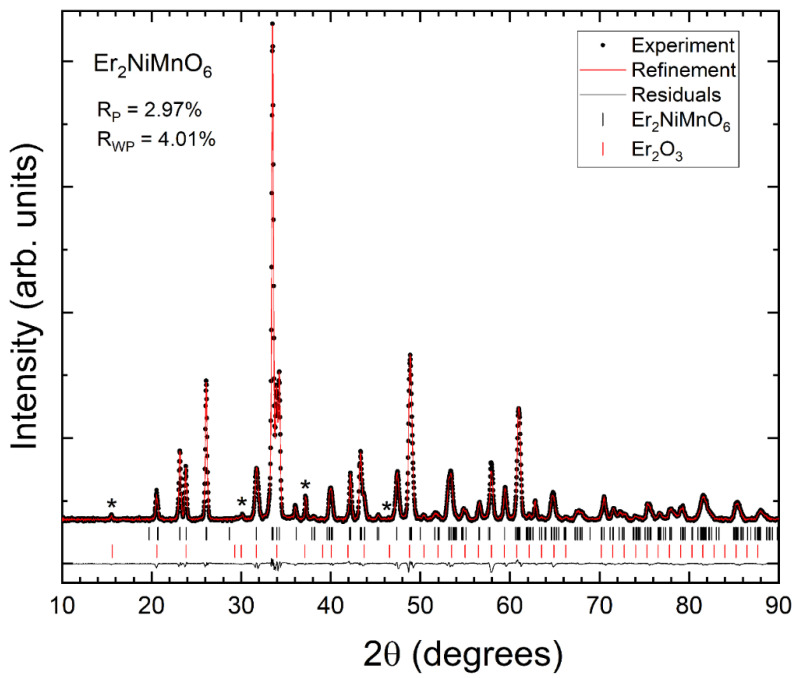
XRD pattern measured in Er_2_NiMnO_6_ (black symbols), Rietveld refinement (red line), and residuals (black line). The ticks show the position of the calculated peaks. R-values are provided in the figure. Asterisks are the most intense peaks of the minority phase.

Powder XRD patterns measured in the three synthesized samples at room temperature are shown in Figure 2, Figure 3 and Figure 4. We found that XRD patterns can be well explained by the known double perovskite structure of R_2_NiMnO_6_ compounds, which is usually described by the non-standard monoclinic space group *P*2_1_/*n* [17]. In addition to the reflection peaks assigned to the studied compounds, we observed extra weak reflections (indicated by asterisks in Figure 2, Figure 3 and Figure 4) that can be assigned to the known bixbyite-type structure of Pr_2_O_3_ (in Pr_2_NiMnO_6_ see Figure 2), of Gd_2_O_3_ (in Gd_2_NiMnO_6_ see Figure 3), and of Er_2_O_3_ (in Er_2_NiMnO_6_ see Figure 4), which is described by space group *Ia*$\bar{3}$ [22,23]. The amount of impurities has been estimated from Rietveld refinements [24] and they are indicated in Table 1. The presence of impurities does not preclude the accurate structural determination of the crystal structure of the studied R_2_NiMnO_6_ compounds. The Rietveld refinements are shown in Figure 2, Figure 3 and Figure 4, including R-values. The refinements show that the assumed structural model for the three studied compounds matches well with the measured XRD patterns. In the refinements, the background was fitted with a Chebyshev polynomial function of the first kind with six coefficients [25]. The shapes of peaks were modelled using a pseudo-Voigt function and a Caglioti model [26]. The unit-cell parameters obtained from the refinements are summarized in Table 2. They agree with the literature [16,17,27]. The atomic positions are summarized in Table 3. The oxygen positions were refined with precision, in spite of the fact that the compounds contain heavy atoms, thanks to the fact that the positions of Ni and Mn are fixed by symmetry.

In Table 2, by comparing the three compounds, it can be seen that there is a monotonic decrease of the unit-cell volume as the size of the rare-earth ion decreases from Pr to Er, which is a consequence of the lanthanide contraction. Table 4 contains some selected bond distances and angles. There it can be seen than in the three compounds the MnO_6_ octahedron is smaller and more regular than the NiO_6_ octahedron. Regarding the coordination polyhedron of the lanthanide atom, in contrast to conventional double perovskites, where the A-site coordination number is 12, in the specific type of R_2_NiMnO_6_, the coordination number of the lanthanide atom is 8, as previously highlighted by Maneesha et al. [16]. Notice that the eight R-O bonds are shorter than 2.7 Å. In the three compounds, the ninth bond is at a distance larger than 3.15 Å. An additional fact to highlight about the coordination of R atoms is that, in the three compounds, there are four distances shorter 2.35 Å and four distances larger than 2.49 Å, which makes the effective coordination number to be close to six. We would like to add here that, according to the calculated Ni-O-Mn angles, the tilting angle $\varphi =\frac{180-<Ni-O-Mn>}{2}$ is close to 17.5° for the three compounds, which is consistent with the fact that the three compounds have a similar β angle. 

From the XRD patterns, we also obtained information on the average particle size using the X’Pert high-score plus v5.2 software and applied both a Langford (L) analysis [28] and the equation proposed by Scherrer applied to the strongest peak [29]. The average crystallite size we determined for each sample is summarized in Table 1. Both methods provide consistent particle sizes, with the Langford method providing slightly smaller sizes.

**Figure 5:**
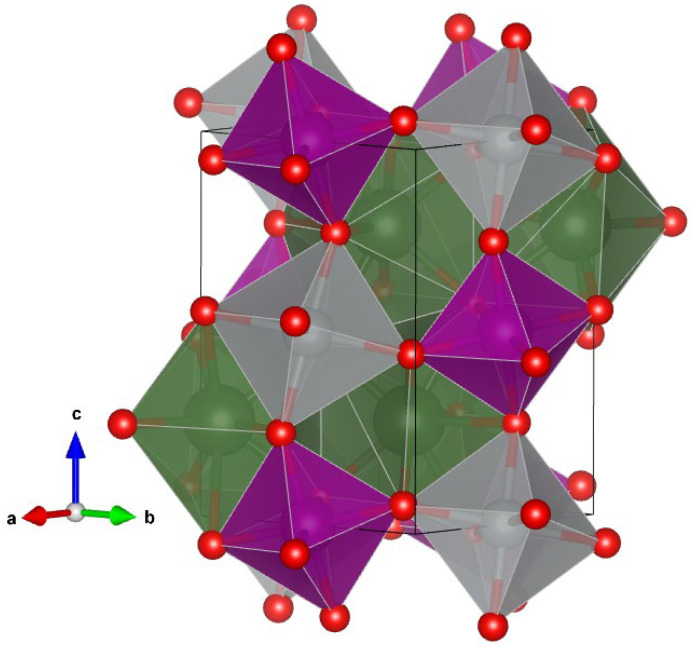
Crystal structure of R_2_NiMnO_6_ compounds. NiO_6_ and MnO_6_ octahedral units are shown in grey and violet, respectively. The RO_8_ dodecahedra are shown in green and the oxygen atoms in red.

A schematic representation of the crystal structure of the three R_2_NiMnO_6_ compounds here studied is shown in Figure 5. The structure can be described as a linear chain of corner-sharing NiO_6_ and MnO_6_ octahedral units running parallel to the c-axis, which are connected by edge-sharing RO_8_ dodecahedra. The NiO_6_ and MnO_6_ units show a significant octahedral tilting. Since NiO_6_ and MnO_6_ units can be considered as uncompressible units in comparison with RO_8_ dodecahedra, the compressibility (and consequently the bulk modulus) of the three studied compounds can be assumed as determined basically by the RO_8_ dodecahedra [30]. Under this hypothesis, and using the empirical formula proposed by Errandonea and Manjon [31], we estimated the bulk modulus (K) of Pr_2_NiMnO_6_, Gd_2_NiMnO_6_, and Er_2_NiMnO_6_ to be 173(5), 176(5), and 179(5) GPa, respectively. The bulk modulus is the measure of how resistant a material is to compression and is very relevant for technological applications, since small changes in the unit-cell volume (and consequently of bond distances) could strongly affect physical properties like the band-gap energy or the magnetic moment. The values here reported for the bulk moduli are very similar to the bulk modulus determined experimentally for La_2_NiMnO_6_, 179(8)–188(28) [32]. It is also comparable to the bulk modulus of perovskite CeScO_3_, 165(7) GPa [33], and the bulk modulus of Ho_2_O_3_, 178(5) GPa [34], two compounds where the bulk modulus is defined by the coordination polyhedron of the lanthanide atom. Both facts support the soundness of our estimations of the bulk modulus, confirming that the coordination polyhedra of the rare-earth atom determines the bulk modulus of the R_2_NiMnO_6_ double perovskite compounds. 

### 3.3. FTIR Measurements

**Figure 6:**
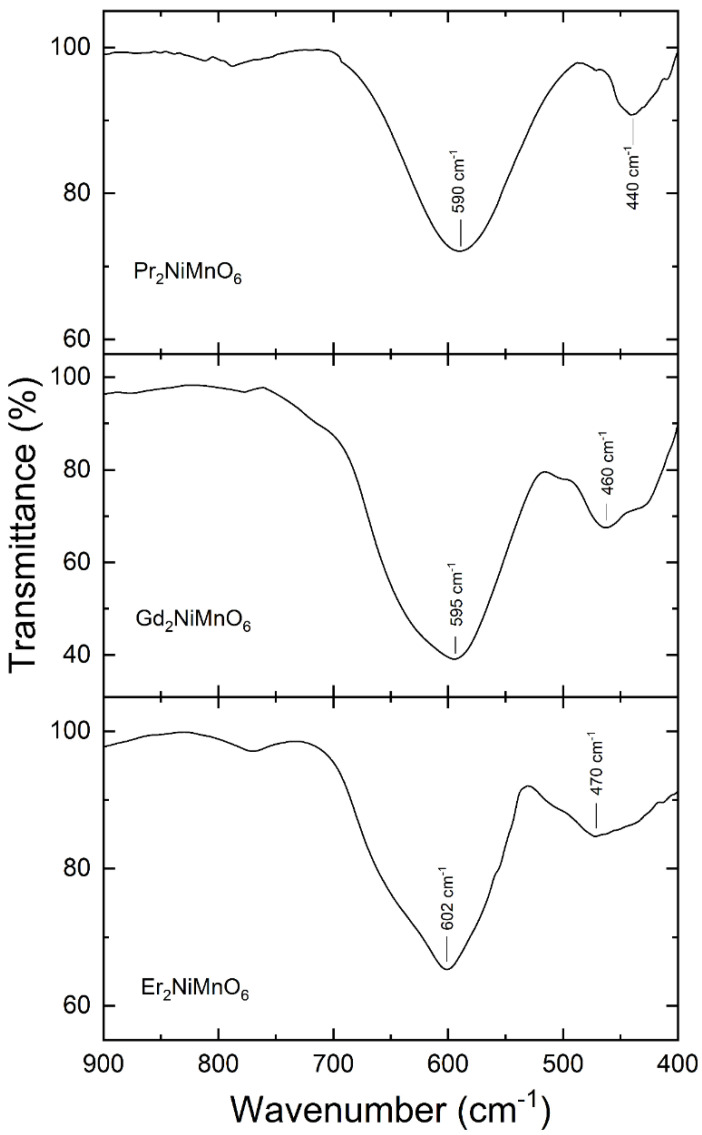
FTIR transmittance spectra measured in the three R_2_NiMnO_6_ double perovskites studied.

Fourier transform infrared spectra were measured in near-normal incidence mode at room temperature in the three studied samples. The FTIR spectra for the samples are shown in Figure 6. They show two strong and well-defined absorption bands. The strongest one has the maximum of the absorption (minimum of transmission) centered at approximately 590–600 cm^−1^ and the other band has it centered at 440–470 cm^−1^. The frequency of the strongest absorption is in the same range that the IR absorption reported for Eu_2_NiMnO_6_ [35] and La_2_NiMnO_6_ [36]. A frequency outside the 590–600 cm^−1^ range was only reported for Sm_2_NiMnO_6_ [37], 575 cm^−1^; probably, this frequency has been underestimated. The frequency of the second absorption is similar to that of the second absorption band reported for La_2_NiMnO_6_ [36]. The frequencies determined from this work are summarized in Table 5. We named the highest and lowest frequency modes as *ν*_1_ and *ν*_2_, respectively.

Having the primitive cell of R_2_NiMnO_6_ compounds two formula units, sixty lattice vibrations are expected. Their mechanical representation is Γ = 12A_g_ + 18A_u_ + 12B_g_ + 18B_u_, where one A_u_ mode plus two B_u_ modes are the acoustic modes. Therefore, at the center of the Brillouin zone there are 24 Raman-active modes (12A_g_ + 12B_g_) and 33 infrared-active modes (17A_u_ + 16B_u_). In previous studies, only two Raman modes and two infrared modes were reported. In La_2_NiMnO_6,_ the reported Raman modes are at 640 and 530 cm^−1^ [37] and the observed infrared modes are redshifted to lower frequencies, 600 and 430 cm^−1^ [38]. The same redshift between Raman and infrared modes is observed when comparing the Raman modes of Pr_2_NiMnO_6_, which are at 657 and 511 cm^−1^ [39], and the infrared modes here reported, which are at 590 and 440 cm^−1^. 

**Figure 7:**
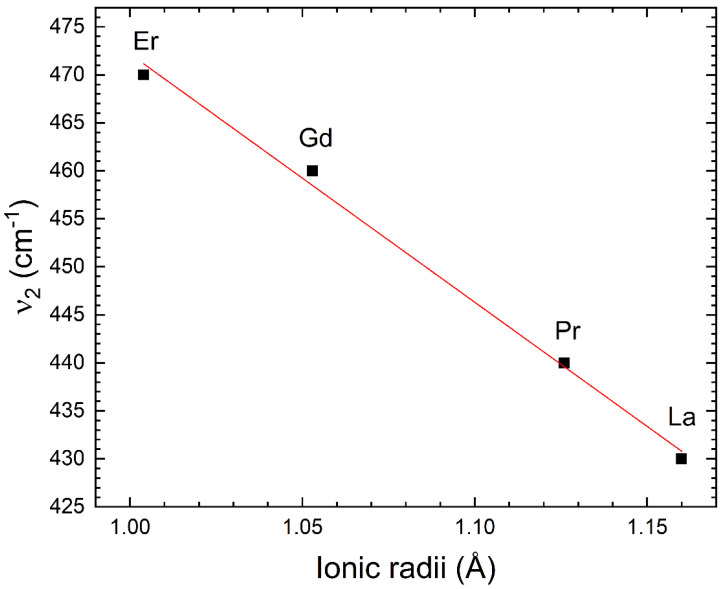
Frequency of the mode ν_2_ versus ionic radii of the lanthanide atom.

The highest frequency Raman and infrared modes have been assigned in the literature to stretching–breathing internal vibrations of the MnO_6_ octahedron [40]. This interpretation is consistent with the fact that the frequency of the ν_1_ mode is basically not affected by the change of the rare-earth atom, as can be seen in Table 5. On the other hand, the frequency of the ν_2_ mode increases as the atomic number increases, showing that this mode is a more complex mode, involving movements of the rare-earth atoms [41]. The increase in the phonon frequency as the mass the lanthanide atom is opposite to the behavior of the Raman modes, where the frequency decreases as the mass of the lanthanide mode increases [42]. Indeed, this is what we would naively expect according to the harmonic approximation, where the frequency is inversely proportional to the square root of the reduced mass of the atoms involved in the vibration [43]. The increase in the frequency of the infrared mode when moving from La to Er along the lanthanide series might be related with the known lanthanide contraction, which makes the ionic radii decrease as the atomic number (and the mass) increases [44]. As a consequence of it, the bond distance within the RO_8_ polyhedron decreases as the atomic mass decreases (see Table 4). Then, if the mode ν_2_ is related to vibrations involving the R-O bonds, an observation of an increase in the frequency of it would be expected, as observed. Note that the reduction in the radius of the lanthanide produces a reduction in the R-O distance and that the force constant of the phonons, and consequently the frequency, which as a first approximation, would follow an inverse relationship with the average R–O bond distance and the ionic radii of the lanthanide atom [43,45] as shown in Table 6 and Figure 7. This figure shows that there is a linear relationship between the frequency of the mode ν_2_ and the ionic radii *r* of the lanthanide element, being $\nu_{2}=730\left(13\right)-258\left(12\right)\times r$. In Table 6, comprehensive estimations of the vibrational frequency ($\nu_{2}$) are presented, specifically focusing on the entirety of the lanthanide family in the context of the remaining R_2_NiMnO_6_ double perovskites, using the equation provided above.

## 4. Conclusions

Double perovskites Pr_2_NiMnO_6_, Gd_2_NiMnO_6_, and Er_2_NiMnO_6_ were synthesized as nanoparticles by a sol–gel method, and a monoclinic crystal structure described by space group *P*2_1_/*n* was identified for them using powder X-ray diffraction and the Rietveld method. Bond distances and angles were determined. The morphology of the samples was studied by electron scanning microscopy, showing that spherical nanoparticles form 100–200 μm agglomerations. The composition was confirmed by energy-dispersive X-ray spectroscopy. The infrared transmittance was measured and the frequencies of the two main phonons determined. Results from infrared spectroscopy were discussed in comparison with previous Raman and infrared studies in related compounds, being the mode assignment discussed. A systematic for the phonon frequencies was discussed and predictions for other R_2_NiMnO_6_ were presented. The bulk modulus of the three compounds was estimated, providing this important parameter for the response of the studied materials to external compression. The synthesis of Pr_2_NiMnO_6_, Gd_2_NiMnO_6_, and Er_2_NiMnO_6_ could open the avenue for additional applications for these materials. The reported results are relevant for energy storage and magnetocaloric applications, and, consequently, to the developing of green technologies. 

## Figures and Tables

**Table 1:** Reagent weights used in reactions, products of synthesis, and particle sizes determined using the Langford (L) and Scherrer (S) methods.

	Ni(NO_3_)_2_·6H_2_O	MnCl_2_·4H_2_O	Product	Particle Size (nm)	
				L	S
Pr(NO_3_)_3_·6H_2_O 0.435 g	0.182 g	0.197 g	Pr_2_NiMnO_6_ 98% Pr_2_O_3_ 2%	37(4)	45(4)
Er_2_O_3_ 0.382 g			Er_2_NiMnO_6_ 94% Er_2_O_3_ 6%	40(4)	49(5)
Gd(NO_3_)_3_·6H_2_O 0.451 g			Gd_2_NiMnO_6_ 98% Gd_2_O_3_ 4%	29(3)	36(3)

**Table 2:** Unit-cell parameters and volume determined for the three synthesized compounds. The estimated bulk modulus (K) is also included.

	Pr_2_NiMnO_6_	Gd_2_NiMnO_6_	Er_2_NiMnO_6_
a (Å)	5.4432(5)	5.2978(5)	5.2302(5)
b (Å)	5.5095(5)	5.6119(5)	5.6392(5)
c (Å)	7.7101(7)	7.5406(7)	7.4533(5)
β (°)	90.17(3)	90.21(3)	90.21(3)
V (Å^3^)	231.2(1) Å^3^	224.2(1) Å^3^	219.8(1) Å^3^
K (GPa)	173(5)	176(5)	179(5)

**Table 3:** Atomic positions obtained from Rietveld refinements for the studied materials.

Pr_2_NiMnO_6_				
	site	x	y	z
Pr	4e	0.9824(5)	0.0705(5)	0.2504(5)
Ni	2d	0.5	0	0
Mn	2c	0.5	0	0.5
O1	4e	0.1074(12)	0.4627(12)	0.2423(12)
O2	4e	0.7009(12)	0.3123(12)	0.0505(12)
O3	4e	0.1783(12)	0.2057(12)	0.9446(12)
Gd_2_NiMnO_6_				
	site	x	y	z
Gd	site	0.9832(5)	0.0713(5)	0.2501(5)
Ni	4e	0.5	0	0
Mn	2d	0.5	0	0.5
O1	2c	0.1082(12)	0.4621(12)	0.2435(12)
O2	4e	0.7016(12)	0.3118(12)	0.0515(12)
O3	4e	0.1771(12)	0.2059(12)	0.9441(12)
Er_2_NiMnO_6_				
	site	x	y	z
Er	site	0.9821(5)	0.0701(5)	0.2512(5)
Ni	4e	0.5	0	0
Mn	2d	0.5	0	0.5
O1	2c	0.1077(12)	0.4330(12)	0.2428(12)
O2	4e	0.7013(12)	0.3133(12)	0.0499(12)
O3	4e	0.1788(12)	0.2051(12)	0.9448(12)

**Table 4:** Selected bond distances and angles.

Lanthanide	Pr	Gd	Er
NiO_6_ octahedron			
Ni-O1 (x2)	2.083(10) Å	2.031(9) Å	2.030(9) Å
Ni-O2 (x2)	2.075(7) Å	2.086(7) Å	2.091(7) Å
Ni-O3 (x2)	2.128(7) Å	2.106(7) Å	2.079(7) Å
MnO_6_ octahedron			
Mn-O1 (x2)	1.967(10) Å	1.933(9) Å	1.936(9) Å
Mn-O2 (x2)	1.969(7) Å	1.942(7) Å	1.924(7) Å
Mn-O3 (x2)	1.938(7) Å	1.945(7) Å	1.952(7) Å
Ni-O-Mn angles			
Ni-O1-Mn	144.3(3)	144.5(3)	144.6(3)
Ni-O2-Mn	146.5(3)	146.4(3)	146.6(3)
Ni-O3-Mn	144.3(3)	144.2(3)	144.1(3)
RO_8_ polyhedron			
R-O1	2.266(8) Å	2.251(7) Å	2.150(8) Å
R-O1	2.311(8) Å	2.292(8) Å	2.281(7) Å
R-O2	2.320(6) Å	2.308(6) Å	2.283(6) Å
R-O2	2.546(7) Å	2.504(7) Å	2.493(7) Å
R-O2	2.677(5) Å	2.630(5) Å	2.603(5) Å
R-O3	2.309(6) Å	2.297(7) Å	2.284(7) Å
R-O3	2.553(7) Å	2.520(7) Å	2.499(7) Å
R-O3	2.696(5) Å	2.640(5) Å	2.614(5) Å

**Table 5:** Frequencies of modes ν_1_ and ν_2_, atomic number of the lanthanide atom, and ionic radii. The frequencies for Eu_2_NiMnO_6_, La_2_NiMnO_6_, and Sm_2_NiMnO_6_ were taken from the literature [35,36,37].

Compound	Z	Ionic Radii (Å)	ν_1_ (cm^−1^)	ν_2_ (cm^−1^)
La_2_NiMnO_6_	57	1.160	600	430
Pr_2_NiMnO_6_	59	1.126	590	440
Sm_2_NiMnO_6_	62	1.079	575	
Eu_2_NiMnO_6_	63	1.066	602	
Gd_2_NiMnO_6_	64	1.053	595	460
Er_2_NiMnO_6_	68	1.004	602	470

**Table 6:** Estimations of the frequencies of $\nu_{2}$ for the lanthanide family hosted in R_2_NiMnO_6_ double perovskites using $\nu_{2}=730\left(13\right)-258\left(12\right)\times r$.

Element	Shannon Ionic Radii. Coord. 8 *	Frequency (cm^−1^)
La	1.160	431(19)
Ce	1.143	435(19)
Pr	1.126	439(19)
Nd	1.109	444(19)
Pm	1.093	448(19)
Sm	1.079	452(19)
Eu	1.066	455(18)
Gd	1.053	458(18)
Tb	1.040	462(18)
Dy	1.027	465(18)
Ho	1.015	468(18)
Er	1.004	471(18)
Tm	0.994	474(18)
Yb	0.985	476(18)
Lu	0.977	478(18)

* Shannon ionic radii from the database of the Atomistic Simulation Group in the Materials Department of Imperial College.

## Data Availability

Data will be made available on request.

## References

[1] Sun H., Chen X.X.G., Zhou Y., Lin H.-J., Chen C.-T., Ran R., Zhou W., Shao Z. (2019). Smart Control of Composition for Double Perovskite Electrocatalysts toward Enhanced Oxygen Evolution Reaction. ChemSusChem.

[2] Xu X., Zhong Y., Shao Z. (2019). Double Perovskites in Catalysis, Electrocatalysis, and Photo(electro)catalysis. Trends Chem..

[3] Oleś M., Horsch P., Feiner L.F., Khaliullin G. (2006). Spin-Orbital Entanglement and Violation of the Goodenough-Kanamori Rules. Phys. Rev. Lett..

[4] Abass S., Bagri A., Sultan K. (2023). Modifications induced in structural, electronic, and dielectric properties of Nd_2_NiMnO_6_ double perovskite by Sr doping. J. Alloys Compd..

[5] Booth R.J., Fillman R., Whitaker H., Nag A., Tiwari R.M., Ramanujachary K.V., Gopalakrishnan J., Lofland S.E. (2009). An investigation of structural, magnetic, and dielectric properties of R_2_NiMnO_6_ (R = rare earth, Y). Mater. Res. Bull..

[6] Jia Y., Cheng Y., Wang H., Zhang Z., Li L. (2020). Magnetocaloric properties and critical behavior in double perovskite RE_2_CrMnO_6_ (RE = La, Pr, and Nd) compounds. Ceram. Int..

[7] Shinde K.P., Lee E.J., Manawan M., Lee A., Park S.Y., Jo Y., Ku K., Kim J.M., Park J.S. (2021). Structural; magnetic, and magnetocaloric properties of R_2_NiMnO_6_ (R = Eu, Gd, Tb). Sci. Rep..

[8] Kumar N., Kaushik S.D., Rao K.S., Babu P.D., Deshpande S.K., Achary S.N., Errandonea D. (2023). Temperature Dependent Crystal Structure of Nd_2_CuTiO_6_: An In Situ Low Temperature Powder Neutron Diffraction Study. Crystals.

[9] Bessimou M., Masrour R. (2024). Study of Optical, Magnetic, and Magnetocaloric Properties of Double Perovskites Dy_2_NiMnO_6_: First Principles Approach and Monte Carlo Simulations. J. Inorg. Organomet. Polym. Mater..

[10] Eerenstein W., Mathur N.D., Scott J.F. (2006). Multiferroic and magnetoelectric materials. Nature.

[11] Singh M.P., Truong K.D., Jandl S., Fournier P. (2011). Magnetic properties and phonon behavior of Pr_2_NiMnO_6_ thin films. Appl. Phys. Lett..

[12] Hashisaka M., Kan D., Masuno A., Takano M., Shimakawa Y., Terashima T., Mibu K. (2006). Epitaxial growth of ferromagnetic La_2_NiMnO_6_ with ordered double-perovskite structure. Appl. Phys. Lett..

[13] Rogado N.S., Li J., Sleight A.W., Subramanian M.A. (2005). Magnetocapacitance and magnetoresistance near room temperature in a ferromagnetic semiconductor: La_2_NiMnO_6_. Adv. Mater..

[14] Li H., Sun L.P., Feng Q., Huo L.H., Zhao H., Bassat J.M., Rougier A., Fourcade S., Grenier J.C. (2017). Investigation of Pr_2_NiMnO_6_-Ce_0.9_Gd_0.1_O_1.95_ composite cathode for intermediate-temperature solid oxide fuel cells. J. Solid State Electrochem..

[15] Guan D., Zhou J., Huang Y.C., Dong C.-L., Wang J.-Q., Zhou W., Shao Z. (2019). Screening highly active perovskites for hydrogen-evolving reaction via unifying ionic electronegativity descriptor. Nat. Commun..

[16] Maneesha P., Baral S.C., Rini E.G., Sen S. (2023). An overview of the recent developments in the structural correlation of magnetic and electrical properties of Pr_2_NiMnO_6_ double perovskite. Prog. Solid. State Chem..

[17] Retuerto M., Muñoz A., Martínez-Lope M.J., Alonso J.A., Mompeán F.J., Fernández-Díaz M.T., Sánchez-Benítez J. (2015). Magnetic Interactions in the Double Perovskites R_2_NiMnO_6_ (R = Tb, Ho, Er, Tm) Investigated by Neutron Diffraction. Inorg. Chem..

[18] Miguel A.S. (2006). Nanomaterials under high-pressure. Chem. Soc. Rev..

[19] Anirban S., Dutta A. (2021). Understanding the structure and charge transport mechanism of Sm_2_NiMnO_6_ double perovskite prepared via low temperature auto-ignition method. Phys. Lett. A.

[20] Yi W., Liang Q., Matsushita Y., Tanaka M., Belik A.A. (2013). High-Pressure Synthesis, Crystal Structure, and Properties of In_2_NiMnO_6_ with Antiferromagnetic Order and Field-Induced Phase Transition. Inorg. Chem..

[21] Zou D., Yi Y., Song Y., Guan D., Xu M., Ran R., Wang W., Zhou W., Shao Z. (2022). The BaCe_0.16_Y_0.04_Fe_0.8_O_3−δ_ nanocomposite: A new high-performance cobalt-free triple-conducting cathode for protonic ceramic fuel cells operating at reduced temperatures. J. Mater. Chem. A.

[22] Saiki A., Ishizawa N., Mizutani N., Kato M. (1985). Structural Change of C-Rare Earth Sesquioxides Yb_2_O_3_ and Er_2_O_3_ as a Function of Temperature. J. Ceram. Soc. Jpn..

[23] Rudenko V.S., Boganov A.G. (1970). Stoichiometry and phase transitions in rare earth oxides. Inorg. Mater..

[24] Rietveld H.M. (1969). A profile refinement method for nuclear and magnetic structures. J. Appl. Crystallogr..

[25] Kaduk J.A. (2009). A Rietveld tutorial-Mullite. Powder Diffr..

[26] Scardi P. (2020). Diffraction Line Profiles in the Rietveld Method. Cryst. Growth Des..

[27] Mohapatra S.R., Sahu B., Raut S., Kaushik S.D., Singh A.K. (2015). Investigation on structural, optical and magnetic properties of double perovskite Gd_2_NiMnO_6_. AIP Conf. Proc..

[28] Langford J.I., Wilson A.J.C. (1978). Scherrer after sixty years: A survey and some new results in the determination of crystallite size. J. Appl. Cryst..

[29] Patterson A. (1939). The Scherrer Formula for X-Ray Particle Size Determination. Phys. Rev..

[30] Errandonea D., Garg A.B. (2018). Recent progress on the characterization of the high-pressure behaviour of AVO_4_ orthovanadates. Prog. Mater. Sci..

[31] Errandonea D., Manjon F.J. (2008). Pressure effects on the structural and electronic properties of ABX_4_ scintillating crystals. Prog. Mater. Sci..

[32] Ridley C.J., Daisenberger D., Wilson C.W., Stenning G.B.G., Sankar G., Knight K.S., Tucker M.G., Smith R.I., Bull C.L. (2019). High-Pressure Study of the Elpasolite Perovskite La_2_NiMnO_6_. Inorg. Chem..

[33] Errandonea D., Santamaria-Perez D., Martinez-Garcia D., Gomis O., Shukla R., Achary S.N., Tyagi A.K., Popescu C. (2017). Pressure Impact on the Stability and Distortion of the Crystal Structure of CeScO_3_. Inorg. Chem..

[34] Garg A.B., Muñoz A., Anzellini S., Sánchez-Martín J., Turnbull R., Díaz-Anichtchenko D., Popescu C., Errandonea D. (2024). Role of GdO addition in the structural stability of cubic Gd_2_O_3_ at high pressures: Determination of the equation of states of GdO and Gd_2_O_3_. Materialia.

[35] Elhamel M., Hebboul Z., Naidjate M.E., Draoui A., Benghia A., Fadla M.A., Kanoun M.B., Goumri-Said S. (2023). Experimental synthesis of double perovskite functional nano-ceramic Eu_2_NiMnO_6_: Combining optical characterization and DFT calculations. J. Solid State Chem..

[36] Ahmad J., Siddique M., Khan J.A., Bukhari S.H., Sultan T. (2019). Impact of rare earth substitution on structural and optical properties of multiferroic La_2−x_Gd_x_NiMnO_6_. Mater. Res. Express.

[37] Mukherjee R., Sheikh M.S., Sinha T.P. (2019). Sintering Temperature Dependent Optical and Vibrational Properties of Sm_2_NiMnO_6_ Nanoparticle. J. Nano-Electron. Phys..

[38] Yang D., Lampronti G.I., Haines C.R.S., Carpenter M.A. (2019). Magnetoelastic coupling behavior at the ferromagnetic transition in the partially disordered double perovskite La_2_NiMnO_6_. Phys. Rev. B.

[39] Truong K.D., Singh M.P., Jandl S., Fournier P. (2011). Investigation of phonon behavior in Pr2NiMnO6 by micro-Raman spectroscopy. J. Phys. Condens. Matter.

[40] Iliev M.N., Guo H., Gupta A. (2007). Raman spectroscopy evidence of strong spin-phonon coupling in epitaxial thin films of the double perovskite La_2_NiMnO_6_. Appl. Phys. Lett..

[41] Ruiz-Fuertes J., Errandonea D., López-Moreno S., González J., Gomis O., Vilaplana R., Manjón F.J., Muñoz A., Rodríguez-Hernández P., Friedrich A. (2011). High-pressure Raman spectroscopy and lattice-dynamics calculations on scintillating MgWO_4_: Comparison with isomorphic compounds. Phys. Rev. B.

[42] Nasir M., Kumar S., Patra N., Bhattacharya D., Jha S.N., Basaula D.R., Bhatt S., Khan M., Liu S.W., Biring S. (2019). Role of Antisite Disorder, Rare-Earth Size, and Superexchange Angle on Band Gap, Curie Temperature, and Magnetization of R_2_NiMnO_6_ Double Perovskites. ACS Appl. Electron. Mater..

[43] Bucknum M.J. (2008). Chemical Physics of Phonons and Superconductivity: A Heuristic Approach. Nat. Preced..

[44] Errandonea D., Boehler R., Ross M. (2000). Melting of the Rare Earth Metals and f-Electron Delocalization. Phys. Rev. Lett..

[45] Liang A., Rahman S., Rodriguez-Hernandez P., Muñoz A., Manjón F.J., Nenert G., Errandonea D. (2020). High-Pressure Raman Study of Fe(IO_3_)_3_: Soft-Mode Behavior Driven by Coordination Changes of Iodine Atoms. J. Phys. Chem. C.

